# Infection parameters of *Heterorhabditis amazonensis* (Nematoda: Heterorhabditidae) in different stages of Hibiscus pink mealybug

**DOI:** 10.21307/jofnem-2020-077

**Published:** 2020-07-21

**Authors:** Yvan Fuenmayor, Edgar Portillo, Brynelly Bastidas, Mayamarú Guerra, Ernesto San-Blas

**Affiliations:** 1 Instituto Venezolano de Investigaciones Científicas, Km 11 Carretera Panamericana, Caracas, Venezuela; 2 Independent Researcher, Gran Avenida 6001, Santiago, Chile; 3 Facultad de Ciencias Básicas, Universidad Tecnológica de Bolívar, Cartagena, Colombia

**Keywords:** Biocontrol, Entomopathogenic nematodes, *Maconellicoccus hirsutus*, Pest control

## Abstract

The pink hibiscus mealybug *Maconellicoccus hirsutus* (Hemiptera: Pseudococcidae) is an invasive pest of an enormous variety of crops and has become a concern in many parts of the world. Early attempts to control *M. hirsutus* with chemical insecticides and cultural methods have failed due to the cryptic habit of the insect. We assessed the entomopathogenic nematode *Heterorhabditis amazonensis* as a biological agent against different insect stages. Comparing different concentrations of the nematode, insect females were very susceptible, with more than 90% of the insects killed. In second and third nymphal stages mortality rates varied from 20 to 60% depending on the nematode concentration. The first nymphal stage as much less susceptible to nematodes due to their small size. The number of nematodes capable of invading the insect host did not vary between the different concentrations. However, the LC50 for females (35.2 IJ/insect), second and third nymphal stages (83.9 IJ/insect) demonstrated that *H. amazonensis* should be considered as a potential biocontrol agent of the pink hibiscus mealybug.

The pink hibiscus mealybug (PHM), *Maconellicoccus hirsutus* Green (Hemiptera: Pseudococcidae), has become a serious pest in Venezuela since its detection in the 1990s. Its host range exceeds 350 botanical species, including fruits, urban trees, ornamentals, and weeds (Kairo et al., 2000; Padilla, 2000; Cermelli et al., 2002). PHM was described in India in 1908, followed by worldwide reports in both subtropical and tropical regions, including Asia, Africa, Northern Australia, the Caribbean, North and South America (Williams, 1996; Padilla, 2000; Chong et al., 2015).

Initial measures to control the pest were done using chemical insecticides and cultural methods but their efficiency was low due to the cryptic zones of the plants where the insects hide and also because their impermeable waxy cover complicates the penetration of insecticides (Sagarra and Peterkin, 1999; Kairo et al., 2000; Chong et al., 2015). For these reasons, biological control by means of parasitoid wasps has been gaining acceptance. Among those natural enemies, *Anagyrus kamali* Moursi (Hymenoptera: Encyrtidae) has shown remarkable results in controlling PHM (Kairo et al., 2000).

In 1996, an initiative proposed by CARICOM (Caribbean Community) and Venezuela was approved to design a long-term sustainable program for control of PHM in the Caribbean region. Over time, most the Caribbean countries developed successful biological control programs (Kairo et al., 2000). Venezuela was an exception because of lack of implementation of insect mass production in laboratories, among other causes (Cermelli et al., 2002). For those reasons, there is a need to study biological alternatives to reduce the populations of PHM in Venezuela.

Entomopathogenic nematodes (EPNs) have been used in biological control programs for crops worldwide because of their effectiveness, time of response, innocuousness to mammals, and relative simplicity of mass production (Kaya et al., 2006). Some laboratories in Venezuela work actively on these organisms (San-Blas et al., 2015), which are currently available in small quantities for biological control programs (San-Blas, Luzardo, Larreal, Portillo and Bastidas, 2017, San-Blas, Luzardo, Portillo, Fuenmayor and Bastidas, 2017) and production of these biocontrol agents is expected to rise shortly (San-Blas et al., 2019).

Currently, the growing interest in controlling hemipterans such as PHM has led to experiments aimed at controlling several species using EPN. For example, *Dysmicoccus vaccinn* Miller and Polavarapu (Hemiptera: Pseudococcidae), *D. texensis* Tinsley, *D. brevipes* Cockerell, *Planococcus citri* Risso (Hemiptera: Pseudococcidae), *P. ficus* Signoret, and *Pseudococcus viburni* Signoret (Hemiptera: Pseudococcidae) were highly susceptible to different species of EPNs (> 90% mortality) in laboratory experiments and under greenhouse conditions (Stuart et al., 1997; Alves and Moino Junior, 2009; Alves et al., 2009; van Niekerk and Malan, 2012; le Vieux and Malan, 2013, 2015; Ferreira et al., 2015; Nomakholwa et al., 2016; Stokwe and Malan, 2017; Guide et al., 2018). As EPNs have shown potential to become biological control agents of the above mentioned hemipterans, the objective of this work was to evaluate the pathogenicity, virulence, and infection rates of *Heterorhabditis amazonensis* Andaló et al. (Rhabditida: Heterorhabditidae) against different stages of PHM under laboratory conditions.

## Materials and methods

### PHM breeding

PHM-Infected fruits of *Annona cherimola* Mill. (Magnoliales: Annonaceae) plants were collected from the field and carried to the laboratory in plastic boxes (1 L). Females were detached from the fruits and ovisacs separated from females. Ovisacs were placed in an Eppendorf tube (1 ovisac per tube) and incubated at 28°C. Daily the tubes were checked until hatching was observed. The new-born nymphs were collected using a small brush and set over a pumpkin (bought from the local market) previously disinfected with 10% sodium hypochlorite and placed in a clean plastic box with a perforated lid (28°C; 75% RH; 12 hr light). The life cycle was monitored every 48 hr and the procedure was repeated when the pumpkin was consumed by the insects. In this stage, all instars were present, so the insects were collected and separated by instars on the day of the experiment.

### Nematode culture

The nematode *H. amazonensis* was cultured in the fourth instar larvae of *Galleria mellonella* (Lepidoptera: Pyralidae) following Dutky et al. (1964) technique. The infected larvae were incubated at 25°C. Infective juveniles were collected in White traps (White, 1927) and stored at 20°C until the experiment proceeded (no more than 2 weeks).

### Virulence, pathogenicity, and mortality determination of different instars of PHM to *Heterorhabditis amazonensis*

Multi-well plates (six wells) were filled with 250 mg sterile sand per well. Randomly, 40 µL of a nematode suspension containing either 0, 10, 40, 60, 80, or 100 IJs/insect were placed over the sand. In every well, five insects were placed and covered with Parafilm^®^ and then aluminum foil. The procedure was repeated for 1st (crawlers), 2nd, and 3rd nymph instars and adult females. The plates were placed in humidity chambers (a plastic box with moistened tissue paper in the bottom) and incubated at 28°C. After 96 hr, the dead insects were counted and dissected. The number of nematodes (J4 and adults) found inside the insect cadavers was recoded to evaluate nematode infection parameters (see below). The experiment was repeated four times. As no mortality was found in the control treatments (concentration = 0), no adjustment of mortality was done.

The percentages of mortality and host infection (number of nematodes found inside the cadaver out of the total nematodes set in the experiment) were angular transformed (arcsine of the square root of the proportions presented in degrees) due to the binomial nature of the data and ANOVA tests were performed to assess differences of host infection of each insect instar at varying IJ concentrations; if differences were statistically significant, a family LSD test was done. Data in figures and tables were presented untransformed (% ± s.e.m.). The penetration rate of infective juveniles per insect was determined using Glazer and Lewis (2000) formulae for different concentrations. The results were angular transformed and treated as above. Data in the figures were presented untransformed (% ± s.e.m.).

To evaluate differences between number of invader nematodes per insect instar and per nematode concentration, the resulting numbers of nematodes found inside the insect cadaver were used untransformed (including 0 values) and a Poisson distribution confirmed (data not shown), followed by a Mood’s median test (Siegel and Castellan, 1988). Data were presented as medians (first quartile, third quartile). Probit calculations were performed to assess lethal concentration of number of nematodes to kill 50 (LD_50_), 75 (LD_75_), and 90 (LD_90_) percent of the insects per instar.

## Results and discussion

**Figure 1:**
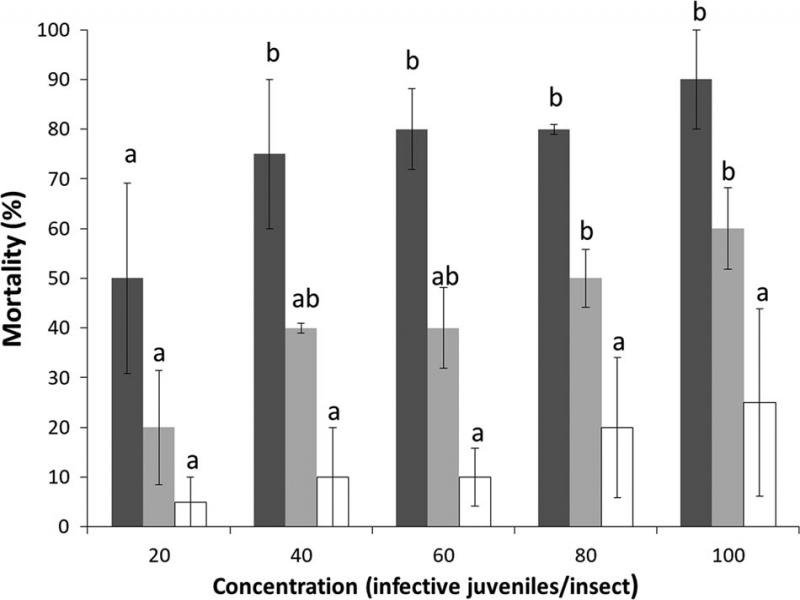
Percentage of mortality of different stages of *Maconellicoccus hirsutus* according to different concentrations of *Heterorhabditis amazonensis*; dark gray bars = females; light gray bars = second and third nymphal stages; white bars = crawlers (first nymphal stage). Bars with the same letters do not differ significantly among treatments per developmental stage (means ± sem).

The mortality rates showed significant variations when the nematode concentration was increased in all PHM instars except in ‘crawlers’ (first nymphal stage) (Fig. 1). The highest mortality was achieved in females (90 ± 10%) using 100 IJs/insect (*F*_(4,15)_ = 8.68; *p* < 0.001). In the second and third nymphal stages, the mortality increased as the nematode concentrations were augmented (*F*_(4,15)_ = 11.54; *p* < 0.001), but the mortality levels were lower than those of females (60 ± 8.16% using 100 IJs/insect). As noted previously, crawlers (first nymphal stage) susceptibility was indifferent to dosage (*F*_(4,15)_ = 0.72; *p* = 0.62).

This difference (developmental stage/nematode concentration) has been observed previously and been used for biological control programs to targeting special insect instars (Schroeder et al., 1996; Kakouli-Duarte et al., 1997). In general, the use of EPN to control other pseudococcids such as *Dysmicoccus. vaccinii*, *D. brevipes*, *Planococcus citri*, and *P. ficus* resulted in higher mortality rates of mature females (Stuart et al., 1997; van Niekerk and Malan, 2012; le Vieux and Malan, 2013; Ferreira et al., 2015).

The variation in mortality rates may be attributed to the size of the instars (Stuart et al., 1997; Bastidas et al., 2014). In our results, the second and third nymphal stages were also susceptible to *H. amazonensis*, but less so compared to adult females. There was also significantly less infectivity of EPNs to the ‘crawlers’. In micro hosts (less than 5 mm), the penetration, development, and reproduction of EPNs are compromised due to a series of factors related to the sizes of both the insect and the nematode (Bastidas et al., 2014).

**Figure 2:**
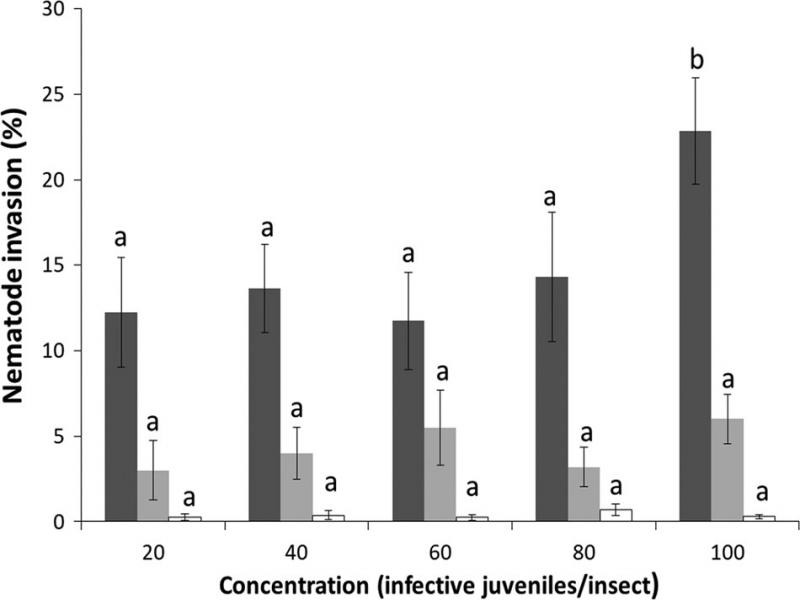
Percent invasion by *Heterorhabditis amazonensis* of different stages of *Maconellicoccus hirsutus* at different concentrations. Dark gray bars = females; light gray bars = second and third nymphal stages; white bars = crawlers (first nymphal stage). Bars with the same letters do not differ significantly among treatments per developmental stage (means ± sem).

In this case, the size of the PHM nymphal stages limited the penetration and infection rates of the nematodes. For example, the penetration rates of *H. amazonensis* against PHM females (Fig. 2) did not vary significantly according to the concentration (*F*_(4,95)_ = 2.16; *p* = 0.079). However, the number of individuals able to invade insects was higher (median of 22 IJ; Q1 = 8; Q3 = 22) when the concentrations increased to 100 IJ/insect (*χ*^2^ = 23.3; d.f. 4; *p* < 0.001) compared to all other concentrations (Table 1). In contrast, the percentage of nematodes able to invade nymphal stages (second and third instars and crawlers) did not show significant differences between the concentrations of nematodes applied to the insects (*F*_(4,95)_ = 1.08; *p* = 0.369 and *F*_(4,95)_ = 0.62; *p* = 0.647, respectively) (Fig. 2). The median number of IJs found inside the host cadavers did not show any relation between the nymphal stages and the concentration (*χ*^2^ = 8.46; d.f. 4; *p* < 0.076 in the second and third instars and *χ*^2^ = 4.49; d.f. 4; *p* < 0.076 for crawlers) and ranged between 4 and 12 individuals per insect (second and third instars) and one IJ per crawler (Table 1). In fact, the number of nematodes capable of penetrating the nymphs was the same even though the EPN concentrations increased. This constant pattern of nematode penetration has been observed previously when the host size is reduced, because there is no available space for more nematodes inside the insect body (Gouge and Hague, 1995; Bastidas et al., 2014).

**Table 1.** Median number of *Heterorhabditis amazonensis* capable to invade different developmental stages of *Maconellicoccus hirsutus*.

*Maconellicoccus hirsutus*				
Stage	Concentration (IJs)	Median (IJs)	First quartile	Third quartile
Females	20	0.5	0	6
	40	6	5	9
	60	4.5	3	10
	80	4.5	3	19
	100	22 a	8	22
2nd and 3rd stages	20	0	0	0
	40	0	2	8
	60	0	5	6
	80	1	0	3
	100	4	5	11
Crawlers	20	0	0	0
	40	0	0	0
	60	0	0	0
	80	1	0	1
	100	1	0	1

**Note:** Different letters mean significant differences (*α* = 0.05).

**Figure 3:**
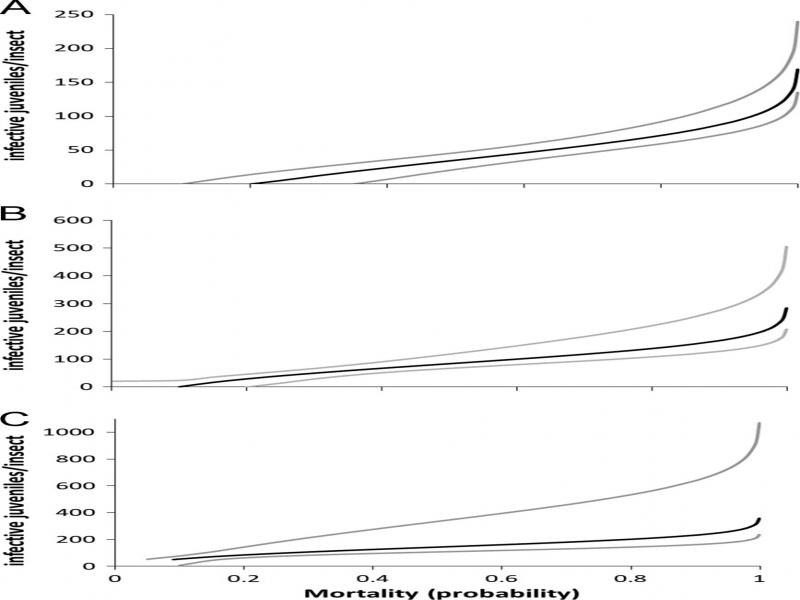
Probability of mortality of different stages of *Maconellicoccus hirsutus* according to the number of *Heterorhabditis amazonensis* (probit). A = females, B = second and third instars, C = crawlers. Black lines represent the mean of the probability. Gray lines represent confidence intervals (95%).

In terms of the potential to use *H. amazonensis* as biological control of PHM, it is evident that females should be the only stage considered to be part of a pest management program using EPN according to the probit models. Because the number of nematodes necessary to kill a given percentage of the insect population must be economically feasible. In our results, the lethal concentrations for females (Fig. 3A) ranged between 34.45 and 91.47 nematodes/insect (LD_50_ and LD_95_ respectively) (Table 2), whereas the number of nematodes required to kill the nymph stages was too high to be considered as biocontrol mean (Fig. 3B, C) (Table 2). The number of *H. amazonensis* required to kill 50 or 95% of the adult females was similar to results reported previously for pseudococcids (van Niekerk and Malan, 2012; le Vieux and Malan, 2013; Ferreira et al., 2015), with the exception of *D. vaccinii* which needed 500 IJ/insect of *H. bacteriophora* to reach 80% of mortality (Stuart et al., 1997).

**Table 2.** Lethal concentrations of *Heterorhabditis amazonensis* to kill different stages of *Maconellicoccus hirsutus*.

Stage	LD_50_	LD_75_	LD_90_
Females	35.2	64.5	106.7
2nd and 3rd stages	83.9	127.6	190.5
Crawlers	142.7	189.7	257.2

The spread and crop damage of PHM is increasing in the tropics, requiring more research aimed at insect control. Some biological control techniques are being successfully used to reduce PHM populations in some countries but EPNs have never been tested. *Cryptolaemus montrouzieri* Mulsant (Coleoptera: Coccinellidae) is an Australian-native ladybird which was successfully released in the Caribbean and India but in Egypt, the beetles are unable to survive winter conditions (Kairo et al., 2000; Mani et al., 2011). Another important biological control agent is the parasitoid *Anagyrus kamali* Moursi (Hymenoptera: Encyrtidae), which has also been released in some Caribbean islands and Egypt with goods results, in the absence of native enemies (Kairo et al., 2000). As EPNs are ubiquitous organisms (Griffin et al., 1990), and have proved to be effective to control some nymphal (second and third) stages and adults females of PHM, EPNs should be considered promptly in integrated pest programs to control this serious pest.
